# Two New Chroman Derivations from the Endophytic *Penicillium* sp. DCS523

**DOI:** 10.3390/molecules16010686

**Published:** 2011-01-18

**Authors:** Jun-Tian Li, Xiao-Li Fu, Chun Tan, Ying Zeng, Qi Wang, Pei-Ji Zhao

**Affiliations:** 1 The State Key Laboratory of Phytochemistry and Plant Resources in West China, Kunming Institute of Botany, Chinese Academy of Sciences, Kunming 650204, China; 2 Engineering Research Center of Chinese Ministry of Education for Edible and Medicinal Fungi, Jilin Agricultural University, Changchun 130118, China; 3 College of Agriculture and Biotechnology, Yunnan Agricultural University, Kunming 650201, China

**Keywords:** chroman derivations, endophytic, *Penicillium* sp., ITS sequence

## Abstract

Strain DCS523 was isolated from the branch tissue of *Daphniphyllum longeracemosum* and determined to be a *Penicillium* sp. according to the ITS sequence analysis. The extracts from the PDA solid fermentation media of *Penicillium* sp. DCS523 were purified to give two new chroman derivatives as well as six known compounds. Based on their spectral data the new compounds were identified as (*Z*)-6-acetyl- 3-(1,2-dihydroxypropylidene)-5-hydroxy-8-methylchroman-2-one (**1**) and 6-acetyl-2α,5- dihydroxy-2-(2-hydroxypropyl)- 3α,8-dimethylchroman (**2**), respectively.

## 1. Introduction

Plant endophytes are a group of microorganisms, including fungi and bacteria, which not only live within plant internal tissues or organs without causing any apparent symptoms or diseases in the host plants, but also serve as important sources of bioactive compounds, presumably due to the symbiotic relationship with their hosts [1]. In the course of our study for chemical constituents from the endophytic microorganisms of plants, a series of new compounds were previously isolated [2,3,4]. In this paper, we investigate the secondary metabolites from *Penicillium* sp. DCS523- an endophytic fungal strain isolated from the surface-sterilized branch of *Daphniphyllum longeracemosum* Rosenth., which is an evergreen tree mainly distributed in Yunnan Province, China [5]. A series of novel daphniphyllines have also been isolated from *D. longeracemosum* [6,7]. Herein, we describe the identification of the strain, and the isolation and structural elucidation of eight compounds, including two new chroman derivatives, from DCS523.

## 2. Results and Discussion

**Figure 1:**
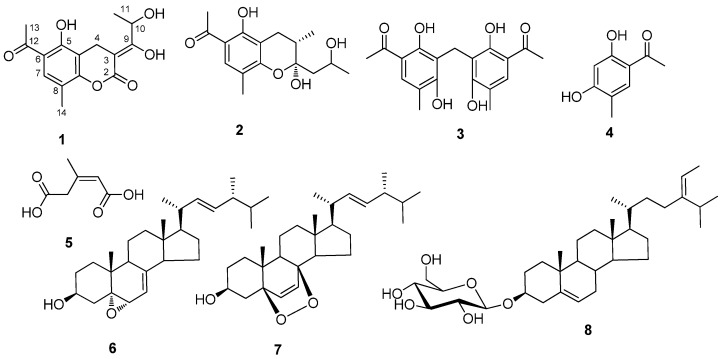
Structures of compounds **1-8**.

The nucleotide sequences for the ITS1-5.8S rDNA-ITS4 region of the endophytic fungi DCS523 was registered in the GenBank database with the accession number HQ179956, and the strain was determined to be *Penicillium* sp. according to the ITS analysis. The chromatographic purification of the extracts from the PDA solid fermentation of *Penicillium* sp. DCS523 provided compounds **1-8** (Figure 1).

Compound **1** was obtained as **a** straw yellow powder. The HR-ESI-MS data indicated a molecular formula of C_15_H_16_O_6_ based on the [*M* + Na]^+^ ion signal at *m/z* 315.0846 (calc. 315.0844). In accordance with the molecular formula, 15 carbon resonances were resolved in the ^13^C-NMR spectrum (Table 1), and were further classified by DEPT experiment into the categories of nine quaternary carbons, two methines, one methylene and three methyls. The structure of **1** was established by detailed HMQC and HMBC experiments (Table 1). The HMBC data showed correlations between H-4 (*δ_H_* 3.53) and the carbons at *δ_C_* 178.6 (C-2), 100.2 (C-3), 114.0 (C-4a) and 161.6 (C-5/8a), between H-7 (*δ_H_* 7.58) and the carbons at *δ_C _*161.6 (C-5/8a), 113.9 (C-6), which suggested the chroman part unit. The other three substituent groups were assigned by following correlations: one methyl was placed at C-8 (*δ_C_* 119.0) on the basis of HMBC correlations from H-14 (*δ_H_* 2.15) to C-7 (*δ_C_* 131.8), C-8 (*δ_C_* 119.0) and C-8a (*δ_C_* 161.6), the acetyl group was assigned at C-6 (*δ_C_* 113.9) on the basis of HMBC correlations from H-13 to C-6 (*δ_C_* 113.9), C-7 (*δ_C_* 131.8) and C-12 (*δ_C_* 204.1), and a 1,2-dihydroxypropylidene group was placed at C-3 (*δ_C_* 100.2) on the basis of HMBC correlations from H-10 (*δ_H_* 4.95) to C-3 (*δ_C_* 100.2), C-9 (*δ_C_* 117.4), and correlations from H-11 (*δ_H_* 1.42) to C-9 (*δ_C_* 117.4) and C-10 (*δ_C_* 76.0). To confirm the lactone connection between the carboxylate group of C-2 to 8a-OH or 5-OH, we recorded the NMR data in DMSO (Table 1), and the result showed the proton (*δ_H _*13.0) of 5-OH was correlated with C-5 (*δ_C_* 161.6) and C-6 (*δ_C_* 113.9). And the NOE correlation between H-11 and H-4 determinated the double bond was Z-configuration. So the compound **1** was determined to be (*Z*)-6-acetyl-3-(1,2-dihydroxypropylidene)-5-hydroxy-8-methylchroman-2-one. 

**Table 1:** NMR data of compound **1** (in CD_3_COCD_3_^ a^ and DMSO^ b^, *J* in Hz).

No.	^1^H*^a^*	^13^C*^a^*	HMBC	^1^H*^b^*	^13^C*^b^*	HMBC
2	-	178.6	-	-	174.8	-
3	-	100.2	-	-	97.1	-
4	3.53 (2H, dd, *J* = 11.8, 23.4 Hz)	15.4	2, 3, 4a, 5, 9	3.37 (2H, brs)	15.0	2, 3, 4a, 5, 9,
4a	-	114.0	-	-	112.1	-
5	-	161.6	-	-	160.8	-
6	-	113.9	-	-	112.1	-
7	7.58 (1H, s)	131.8	5, 6, 8, 8a, 12,14	7.53 (1H, s)	130.9	5, 6, 8, 8a, 12,14
8	-	119.0	-	-	116.3	-
8a	-	161.6	-	-	161.1	-
9	-	177.4	-	-	176.8	-
10	4.95 (1H, d, *J* = 6.7 Hz)	76.0	3, 9, 11	4.78 (1H, q, 6.4)	73.9	3 (w), 9, 11
11	1.42 (3H, d, *J* = 6.8 Hz)	17.8	9, 10	1.31 (3H, d, 6.8)	17.9	9, 10
12	-	204.1		-	203.2	-
13	2.56 (3H, s)	26.3	6, 7, 12	2.51 (3H, s)	26.2	6, 7, 12
14	2.15 (3H, s)	16.0	7, 8, 8a	2.11 (3H, s)	16.2	7, 8, 8a,
5-OH	-	-	-	13.0 (1H, s)	-	5, 6

Compound **2** was obtained as a straw yellow powder. The HR-ESI-MS data indicated a molecular formula of C_16_H_22_O_5_ based on the [*M* + Na]^+^ ion signal at *m/z* 317.1365 (calc. 317.1364). The NMR data (Table 2) were similar to those of compound **1**, and according to the NMR data compound **2** was also a chroman. The detailed structure was elucidated by 2D-NMR. The HMBC data showed correlations between H-3 (*δ_H_* 2.08 ) and the carbons at *δ_C_* 102.4 (C-2), 24.7 (C-4), 109.5 (C-4a) and 161.9 (C-5), between H-4 (*δ_H_* 2.34 and 2.92) and the carbons at *δ_C_* 102.4 (C-2), 34.5 (C-3), 109.5 (C-4a), 158.6 (C-8a), and between H-7 (*δ_H_* 7.53) and the carbons at *δ_C_* 161.9 (C-5), 158.6 (C-8a), which elucidated the chroman part unit. Three substituent groups (5-OH, 6-acetyl and 8-methyl) positions at benzene ring were the same as those of compound **1** (Table 1 and Table 2). The other methyl was placed at C-3 (*δ_C_* 34.5) on the basis of HMBC correlations from H-12 (*δ_H_* 0.94) to C-2 (*δ_C_* 102.4), C-3 (*δ_C_* 34.5) and C-4 (*δ_C_* 24.7). And the HMBC correlations between H-9 (*δ_H_* 1.77 and 1.98) and the carbons at *δ_C_* 102.4 (C-2), *δ_C_* 65.4 (C-10) and *δ_C _*25.0 (C-11), between H-11 (*δ_H_* 1.25) and the carbons at *δ_C_* 42.1 (C-9) and *δ_C_* 65.4 (C-10) to determinate the 2-hydroxypropyl position. The NOE correlations between H-9α (*δ_H_* 1.77) with 2-OH (*δ_H_* 7.07) and H-12 (*δ_H_* 0.94), suggested 2-OH and 12-Me are same orientation, so the structure of **2** elucidated as 6-acetyl-6-acetyl-2α,5-dihydroxy-2-(2-hydroxy- propyl)-3α,8-dimethylchroman.

Compounds **3-5** were identified as 5:5’-diacetyl-2:6:2’:6‘-tetrahydroxy-3:3’-dimethyl- diphenylmethane (**3**) [8], 2,5-dihydroxy-4-methylacetophenone (**4**) [9], *Z*-3-methylpent-2-en-1,5-dioic acid (**5**) [10] by comparison with the data given in references respectively. The three known sterols were identified as 5α,6α-epoxy-24(*R*)-methylcholesta-7,22-dien-3β-ol (**6**) [11], 5α,8α-epidioxy- ergosta-6,22-dien-3β-ol (**7**) [12], and 3β-*O*-D-glucosylstigmasta-5,24(28)-diene (**8**)[13] on the basis of their NMR data and comparison with the data given in references.

**Table 2:** NMR data of compound **2** (in CD_3_COCD_3_, *J* in Hz).

No.	^1^H	^13^C	HMBC
2	-	102.4	-
3	2.08 (1H, m)	34.5	4a, 2,4, 12
4	2.92 (1H, m)	24.7	2, 3, 4a, 5, 7(w) , 8a, 12
	2.34 (1H, dd, *J* = 6.8, 16.8 Hz)		2, 3, 4a, 5, 7(w) , 8a, 12
4a	-	109.5	-
5	-	161.9	-
6	-	113.3	-
7	7.53 (1H, s)	130.6	13, 5, 8a, 8, 6, 4a, 15
8	-	117.7	-
8a	-	158.6	-
9	1.98 (1H, brd, 13.8)	42.1	2, 10
	1.77 (1H, dd, *J* = 10.8, 15.0)		2, 10
10	4.58 (1H, m)	65.4	-
11	1.25 (3H, d, *J* = 6.3 Hz)	25.0	9, 10
12	0.94 (3H, d, *J* = 7.2 Hz)	16.1	2, 3, 4
13	-	204.0	-
14	2.52 (3H, s)	26.4	6, 7 (w) ,13
15	2.13(3H, s)	15.7	7, 8a, 8
5-OH	13.0 (1H, s)	-	4a, 5, 6
2-OH	7.07 (1H, s)	-	2, 3, 9
10-OH	4.93 (1H, s)	-	9, 10

No inhibitory activity was observed for compounds **1** and **2** against the cell lines HL-60, SMMC-7721, MCF-7, A-549, and SW480 at 40 μM in the MTT assays.

## 3. Experimental

### 3.1. General

Optical rotations were measured with a Jasco DIP-370 digital polarimeter. UV spectra were measured on a Shimadzu UV-2401PC spectrophotometer, *λ*_max_ (log *ε*) in nm. NMR spectra were obtained with Bruker AM-400, Bruker DRX-500 and Bruker AVANCE III-600 spectrometers with TMS as internal standard. ESIMS and HRESIMS were recorded on Finnigan LCQ-Advantage and VG Auto-Spec-3000 mass spectrometers, respectively. Column chromatography was performed on silica gel G (200-300 mesh) and H (Qingdao Marine Chemical Factory, China), and Sephadex LH-20 (Amersham Pharmacia, Sweden). HPLC: Waters series HPLC 2695 (Waters Corporation).

### 3.2. Microbial Material

Branch tissue of *D. longeracemosum* was collected at Kunming Botanical Garden, Kunming Institute of Botany, The Chinese Academy of Sciences, Yunnan, China, in August 2009. The plant materials were washed with running tap water and sterilized successively with 75% ethanol for 1 min and 0.1% corrosive sublimate for 5 min, then rinsed in sterile water for five times and cut into small pieces. These small pieces were incubated at 26 °C on PDA media (potato 200 g, dextrose 20 g, agar 15 g, distilled water 1,000 mL) and cultured until colony or mycelium appeared surrounding the segments. After culturing about two weeks, a strain named DCS523 appeared and was isolated from the sterilized branch. It was deposited at the Kunming Institute of Botany, Chinese Academy of Sciences, Kunming, China.

**Figure 2:**
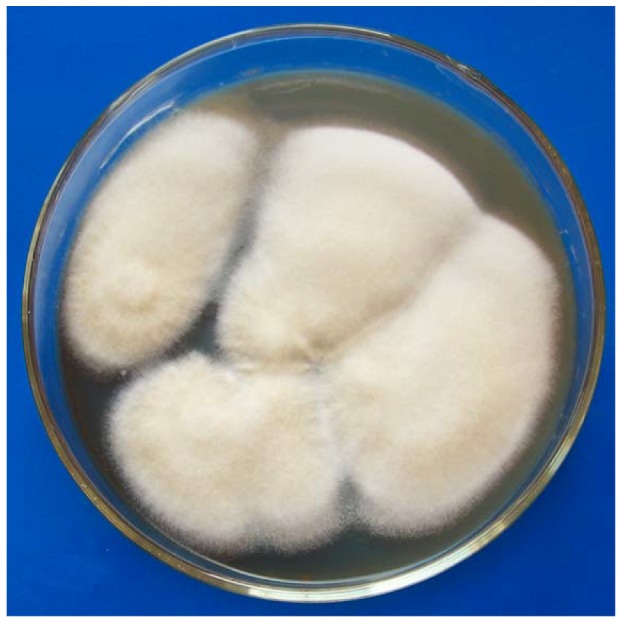
Picture of *Penicillium* sp.DCS523.

### 3.3. Identification of DCS523 by Amplification of the *5.8S* rRNA Gene

The total DNA of DCS523 was extracted by wrapper methods [14]. PCR was performed in a total volume of 50 μL using Primers ITS1 (5’> TCC GTA GGT GAA CCT GCG G <3’) and ITS4 (5’> TCC TCC GCT TAT TGA TAT GC <3’). Amplification reaction mixture contains 100 ng DNA template, 0.5 μM primers, 0.2 mM dNTP, l×Ex-Taq buffer (Takara), and 1.25 U of Ex-Taq (Takara). The reaction mixture was incubated in a thermal cycler (Eppendorf) as follows: 10 min of predenaturation; then 25 cycles of 1 min of denaturation at 94 °C, annealing at 54 °C for 1 min, and elongation for 1.5 min; 10 min of additional extension at 72 °C. The 600 pb PCR products were recovered by gel purification using UNIQ-10 column DNA gel extraction kit (Shanghai Sangon Biotechnology Co., Ltd) and ligated into pUCm T vector (Sangon). The competent *E. coli* JM109 was prepared and plasmids were transformed into it by standard method [14]. Three randomly picked clones were sequenced by ABI 3700 sequencer for insert fragment. The 5.8S *rRNA* partial sequence was assembled using Vector NTI software and blasted against the latest GenBank database using BLASTn.

### 3.4. Extraction and Isolation

The strain was cultured on PDA solid medium (10 L). After three weeks, the cultures were extracted exhaustively five times with EtOAc-MeOH-AcOH (80:15:5, v/v/v) to obtain the corresponding extracts. The extracts (14 g) were chromatographed on silica gel (silica gel G, 80 g) and eluted with petroleum ether/acetone (10:1 to 7:3) and then chloroform/methanol (20:1 to 8:2) to afford six fractions (Fr-1 to Fr-6). Fr-2 (660 mg) was applied to silica gel (silica gel H, 12 g) and eluted with petroleum ether/chloroform (10:1 to 1:1) and then purified by HPLC to obtain compound **3** (2.5 mg). Fr-3 (266 mg) was subjected to silica gel chromatography (silica gel H, 15 g) eluted with petroleum ether/ethyl acetate (50:1 to 8:2) to afford three fractions (Fr3-1 and Fr3-3). Fr3-1 (60 mg) was applied on Sephadex LH-20 eluting with chloroform/methanol (1:1) to obtain compound **4** (6 mg). Fr3-2 (80 mg) was chromatographed on silica gel (silica gel H, 15 g) and eluted with petroleum ether/ethyl acetate (20:1) to afford compound **7** (4.4 mg). Fr3-3 (100 mg) was purified on Sephadex LH-20 eluting with acetone and further purified by HPLC to obtain compound **2** (2.7 mg). Fr-4 (3.0 g) was chromatographed on silica gel (silica gel G, 40 g) and eluted with petroleum ether/acetone (10:1 to 7:3) to chloroform/methanol (10:1 to 8:2) to produce three fractions (Fr4-1 to Fr4-3). Fr4-2 (130 mg) was subjected to silica gel column chromatography (silica gel G, 8 g) eluting with petroleum ether/acetone (20:1 to 10:1) to afford compound **1** (4 mg). Fr4-3 (18 mg) was applied on a silica gel column (silica gel H, 4 g) eluting with petroleum ether/acetone (10:1 to 4:1) to give compound **6** (5.4 mg). Fr-5 (3.5 g) was chromatographed on silica gel (silica gel G, 40 g) eluted with chloroform/methanol (10:1 to 7:3) and then purified on silica gel (silica gel G, 15 g) and eluted with chloroform/acetone (7:3) to obtain compound **8** (10 mg). Fr-6 (3.0 g) was chromatographed on silica gel (silica gel G, 45 g) and eluted with chloroform/methanol (10:1 to 1:1), and rechromatographed on Sephadex LH-20 (30 g) eluting with methanol and then purified on HPLC to obtain compound **5** (10 mg).

*(Z)-6-Acetyl-3-(1,2-dihydroxypropylidene)-5-hydroxy-8-methylchroman-2-one* (**1**). Straw yellow powder; 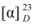 = -5.79 (*c* = 0.16, acetone); UV (CHCl_3_) λ_max _(log ε): 194.6 (5.16), 218.6 (5.28), 259.4 (5.27), 292 (4.92), 327.4 (4.85); for NMR data see Table 1; ESI-MS: 315 ([*M* + Na]^+^), 291 ([*M* - H]^-^); HR-ESI-MS: 315.0846 (([*M* + Na]^+^), calc. 315.0844).

*6-Acetyl-2α,5-dihydroxy-2-(2-hydroxypropyl)-3α,8-dimethylchroman* (**2**). Straw yellow powder. 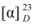 = -19.06. (*c* = 0.12, acetone); UV (CHCl_3_) λ_max _(log ε): 216.4 (5.23), 283.6 (5.04), 328.8 (4.67); for NMR data see Table 2; ESI-MS: 317 ([*M* + Na]^+^), 293 ([*M* - H]^-^); HR-ESI-MS: 317.1365 ([*M* + Na]^+^), calc. 317.1364).

*5:5’-Diacetyl-2:6:2’:6‘-tetrahydroxy-3:3’-dimethyldiphenylmethane* (**3**). Colorless powder. ^1^H-NMR (400 MHz, CDCl_3_) δ: 2.17 (6H, s), 2.57 (6H, s), 1.04 (2H, s), 7.41 (2H, s); ^13^C-NMR (100 MHz, CDCl_3_) δ: 15.9 (CH_2_), 16.1 (CH_3_), 25.9 (CH_3_), 112.2 (C), 112.5 (C), 118.7 (C), 130.9 (CH), 159.0 (C), 161.3 (C), 203.2 (C); EI-MS: 344 ([*M*]^+^). 

*2,5-Dihydroxy-4-methylacetophenone* (**4**). Colorless powder; ^1^H-NMR (500 MHz, CD_3_OD) δ: 2.19 (3H, s), 2.55 (2H, s), 6.33 (1H, s), 7.46 (1H, s);^ 13^C-NMR (125 MHz, CD_3_OD) δ: 15.5 (5-Me), 26.2 (1-COCH_3_), 102.8 (C-3), 114.0 (C-4), 118.2 (C-1), 134.1 (C-6), 164.5, 164.7, 204.1(1-COCH_3_); ESI-MS: 165 ([*M* - H]^-^). 

*(Z)-3-Methylpent-2-en-1,5-dioic acid* (**5**). Colorless powder; ^1^H-NMR (500 MHz, CD_3_OD) δ: 1.96 (3H, s), 3.67 (2H, s), 5.84 (1H, s);^ 13^C-NMR (125 MHz, CD_3_OD) δ: 25.8 (3-Me), 39.5 (C-4), 120.5 (C-2), 152.5 (C-3), 169.9 (C-1), 174.3 (C-5); ESI-MS: 143 ([*M* - H]^-^), 167 ([*M* + Na]^+^). 

*5α,6α-Epoxy-24(R)-methylcholesta-7,22-dien-3β-ol* (**6**). Colorless powder; 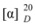 = -48.0 (*c* 0.34, CHCl_3_); EI-MS: 412 [M]^+^; the NMR data were same as reported in the literature [11].

*5α,8α-Epidioxyergosta-6,22-dien-3β-ol* (**7**). Colorless powder. 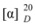 = -55 (*c* 0.4, CHCl_3_);. ESI-MS: 429 [M + H]^+^; the NMR data were the same as in reference [12].

*3β-O-D-Glucosylstigmasta-5,24(28)-diene* (**8**). Colorless powder; ESI-MS: 597 ([*M* + Na]^+^); the NMR data were the same as reported in the literature [13].

## 4. Conclusions

Two new chromans as well as six known compounds were isolated from an endophytic *Penicillium* sp. DCS523. The new compounds were determined by spectroscopic methods, including HR-ESI-MS and 2D-NMR experiments, to be (*Z*)-6-acetyl-3-(1,2-dihydroxypropylidene)-5-hydroxy-8-methyl- chroman-2-one (**1**) and 6-acetyl-2α,5-dihydroxy-2-(2-hydroxy- propyl)-3α,8-dimethylchroman (**2**). 
